# The impact of coronavirus disease 2019 (COVID-19) on older adults with an intellectual disability during the first wave of the pandemic in Ireland

**DOI:** 10.12688/hrbopenres.13238.1

**Published:** 2021-08-19

**Authors:** Mary McCarron, Darren McCausland, Retha Luus, Andrew Allen, Fintan Sheerin, Eilish Burke, Eimear McGlinchy, Fidelma Flannery, Philip McCallion

**Affiliations:** 1 Trinity Centre for Ageing and Intellectual Disability, Trinity College Dublin, Dublin, Ireland; 2 School of Nursing and Midwifery, Trinity College Dublin, Dublin, Ireland; 3 School of Social Work, Temple University, Philadelphia, USA

**Keywords:** Intellectual disability, ageing, COVID-19, health, well-being

## Abstract

**Background:** People with intellectual disability have increased risk of exposure to and adverse outcomes from coronavirus disease 2019 (COVID-19).They also face challenges to mental health and well-being from COVID-19-related social restrictions and service closures.

**Methods:** Data from a supplemental COVID-19 survey from the Intellectual Disability Supplement to the Irish Longitudinal Study on Ageing (IDS-TILDA) (n=710) was used to assess outcomes from the first infection wave of COVID-19 among adults with intellectual disability aged 40+ years in Ireland. Data was gathered on testing, for symptoms and outcomes; procedures to manage COVID-19; and both stress/anxiety and positive experiences during the pandemic. Demographic and health-related data from the main IDS-TILDA dataset was included in analyses.

**Results:** High rates were identified of health conditions associated with poorer COVID-19 outcomes, including overweight/obesity (66.6%, n=365), high cholesterol (38.6%, n=274) and cardiovascular disease (33.7%, n=239). Over half (53.5%, n=380) reported emotional, nervous or psychiatric disorders. Almost two-thirds (62.4%, n=443) were tested for COVID-19, with 10% (n=71) reporting symptoms and 2.5% (n=11) testing positive. There were no instances of COVID-19 related mortality. Common symptoms included fatigue, fever, and cough. Some participants (7.8%, n=55) moved from their usual home to isolate, most often (n=31) or relocate to a family home (n=11). Three-quarters (78.7%) of those who were symptomatic or who tested positive had plans to manage self-isolation and two-thirds were able to comply with guidelines. Over half (55%, n=383) reported some COVID-19 related stress/anxiety; and a similar proportion reported positive aspects during this period (58%, n=381).

**Conclusions:** Our data suggests that people with intellectual disability avoided the worst impacts of COVID-19 during the first infection wave in Ireland. Nevertheless, participants’ health profiles suggest that this population remains at high risk for adverse infection outcomes. Repeated measures are needed to track health and well-being outcomes across multiple infection waves.

## Introduction

Since first being identified, our understanding of coronavirus disease 2019 (COVID-19) including its impact on people with intellectual disabilities (ID) has evolved rapidly following an unprecedented focus from the international scientific community.

### Risk factors of COVID-19

Research to date has identified that people whose living or working circumstances require them to be in proximity or contact with others such as healthcare workers have an increased exposure and therefore increased risk of contracting the disease ( Health Protection Surveillance Centre, 2020; Nguyen *et al*., 2020). People working in other industries with environmental and socio-economic conditions conducive to spreading the disease also have increased risk, for example in the meat processing and mining industries ( Donahue *et al*., 2020; Durand-Moreau *et al*., 2020; Jones, 2020).

Similarly, residents of congregated care settings such as nursing homes, and other long-term care facilities - were also identified as having increased risk of contracting COVID-19 ( Centers for Disease Control and Prevention, 2020; Department of Health (Australia), 2020; European Centre for Disease Prevention and Control, 2020; Government of Canada, 2020; Health Service Executive, 2020).

### Risk and people with intellectual disability

In Ireland, people with intellectual disability were included in the second tier of vulnerable, high-risk groups for severe outcomes of COVID-19, but not ‘extremely vulnerable’ or very high-risk ( Health Service Executive, 2020). The United Kingdom (UK) initially did not classify people with intellectual disability as higher risk, but later added all adults with Down syndrome to the list of ‘clinically extremely vulnerable groups’ ( Public Health England, 2020b). Elsewhere, while not classifying people with intellectual disability as high-risk, the United States (US) identified intellectual disability as a factor which may require extra preventative precautions ( Centers for Disease Control and Prevention, 2020). Similarly, Australia issued additional advice for people with disabilities without including them among high-risk groups ( Department of Health (Australia), 2020); and Canada identified people in group residences and people with reduced capacity as more exposed to COVID-19, without specifying people with intellectual disability as ‘vulnerable’ ( Government of Canada, 2020).

Some of the health conditions previously associated with poorer outcomes for COVID-19 are more prevalent in the intellectual disability population, for example diabetes and obesity ( McCarron *et al*., 2013; McCarron *et al*., 2017a; Shireman *et al*., 2010). Prior to the COVID-19 pandemic, data from the UK suggested that people with intellectual disability were twice as likely to die from causes that may be avoidable with good quality healthcare ( Heslop *et al*., 2014). A recent US study found that the leading cause of death for adults with intellectual disability was heart disease, the same as people without intellectual disability. However, adults with intellectual disability had higher risk of death from diabetes mellitus, dementia, pneumonitis and influenza/pneumonia ( Landes *et al*., 2021).

People with intellectual disability experience the onset of some age-related health conditions at a younger age ( Burke *et al*., 2014), some of which are associated with increased risk for COVID-19. In a 2017 study among adults with intellectual disability aged 40+ years, those with mild intellectual disability (88%) and women (83%) had particularly high rates of obesity; over half (52%) reported a diagnosis of a mental health disorder, including anxiety (32%), depression (16%) and mood swings (15%); many had mobility difficulties, with almost half (45.2%, 295/652) reporting difficulty walking 100 yards and almost a third (31.5%, 209/664) difficulty walking across a room; and people with Down syndrome had higher risk of dementia (36%) ( McCarron *et al*., 2017b). People with Down syndrome also experience long-term dysregulation of their immune system, which may be another added risk for this population ( Espinosa, 2020).

***Increased risk of adverse COVID-19 outcomes***. In public health guidance, people aged 70 years or over and those with specific medical conditions (for example, organ replacement, cancer, severe cystic fibrosis or severe respiratory conditions) are in the highest risk categories; while increasing age generally and the presence of less severe health conditions also raises the risk of adverse COVID-19 outcomes ( Centers for Disease Control and Prevention, 2020; Department of Health (Australia), 2020; Government of Canada, 2020; Health Service Executive, 2020; National Health Service, 2020).

In the general population mortality rates associated with COVID-19 increase exponentially above the age of 50 years, from <1.1% in patients aged under 50 years up to 29.6% in patients aged ≥80 years, with age >60 years identified as a key threshold ( Bonanad *et al*., 2020). However, other studies identified that age alone may not be the most significant factor, with comorbid health conditions including cardiovascular disease (CVD), hypertension, obesity, diabetes, chronic obstructive pulmonary disease (COPD), dementia, depression and chronic kidney disease (CKD) associated with increased mortality and other adverse outcomes ( Aggarwal *et al*., 2020; Carrillo-Vega *et al*., 2020; Hashim *et al*., 2020; Nandy *et al*., 2020).

Older people living in congregated care settings are reported to experience a multiplier risk effect, being (a) more exposed to the virus in their living/care environment, and (b) more vulnerable to severe outcomes due to age and increased comorbidities ( Kennelly *et al*., 2021).

***Outcomes of COVID-19 infection for people with intellectual disability***. Data from the Netherlands found a mortality rate of 11% among people with intellectual disability with a confirmed COVID-19 infection ( Sterker Op Eigen Benen, 2020b). A US study reported higher mortality rates in adults with intellectual and developmental disability (IDD) in the 18–75 year age group compared to those without IDD ( Turk *et al*., 2020), indicating greater mortality risk at a younger age for people with IDD. Two UK studies estimated the adjusted COVID-19 mortality rate for people with intellectual disability was three to eight times higher ( Watkins, 2020) and 6.3 times higher ( Public Health England, 2020a) than the general population, and a 10-fold increased mortality risk was estimated for people with Down syndrome ( Clift *et al*., 2020). Another UK study identified significantly higher rates of mortality from COVID-19 in people with Down syndrome, especially from age of 40 ( Hüls *et al*., 2020).

Higher mortality rates for people with intellectual disability were evident among younger age cohorts ( Public Health England, 2020a). The LeDeR Programme (2020) found that, whereas almost half (47%) of COVID-19 deaths in the general population were among people aged 85 years or more, just 4% of COVID-19 deaths among people with intellectual disability were in this 85+ years category. Perera *et al*. (2020) found a younger mean age of COVID-19 related death (64 years) among people with intellectual disability compared with the general population, while average age of death from COVID-19 was 51 years for people with Down syndrome ( Hüls *et al*., 2020).

Heslop (2020) reported increased mortality risk with epilepsy and found that one in five cases reviewed were discharged from hospital but readmitted soon afterwards; while also identifying risk of infection with mobility impairments and/or mental health needs ( Heslop, 2020). A review of 66 deaths observed high rates of moderate-to-profound intellectual disability (n=43), epilepsy (n=29), mental illness (n=29), dysphagia (n=23), Down syndrome (n=20) and dementia (n=15) ( Perera *et al*., 2020).

Clift *et al.* (2020) found that cardiovascular and pulmonary diseases and care home residence explained some but not all of the increased risk for people with Down syndrome. Hüls *et al.* (2020) found that the main symptoms of COVID-19 for people with Down syndrome (fever, cough and shortness of breath) and risk factors for severe outcomes (older age, male, diabetes, obesity, dementia) were similar to the general population although presented at a younger age. In the Netherlands, nearly two-thirds (62%) of confirmed COVID-19 infections in people with intellectual disability were in the 40–69 age group; while the majority of infections were among those who lived in a group home (83%), with 17% living in their own apartment ( Sterker Op Eigen Benen, 2020a).

Studies conducted early in the pandemic suggested that many people had experienced psychological effects including anxiety and depression and self-reported stress that may have been associated with disturbed sleep ( Rajkumar, 2020), with one study estimating that one-fifth had experienced severe/very severe anxiety ( Moghanibashi-Mansourieh, 2020). A global survey with 8,000 participants, half of whom were healthcare professionals, found that almost one-third had suicidal thoughts, healthcare professionals reported more depression and anxiety, and people with pre-existing suicidal thoughts were less likely to communicate or engage in coping strategies ( Rathod *et al*., 2020). A UK panel study with 55,000 participants associated changes in activities during the pandemic with changes in mental health and wellbeing ( Bu *et al*., 2020). A study of children and adolescents found that the pandemic increased restlessness, irritability, anxiety, clinginess and inattention, which were associated with increased screen time during quarantine ( Imran *et al*., 2020).

The Irish Longitudinal Study on Ageing (TILDA) confirmed an increase in perceived stress and a substantial increase in depressive symptoms among the general older population in Ireland aged 60 years and above during the pandemic (July-November 2020). TILDA reported 65% of participants with perceived stress (36% moderately stressed; 29% most stressed), 27% with anxiety (16% mild; 8% moderate; 3% severe) and 21% with clinically significant depressive symptoms ( De Looze & McDowell, 2021). Older residents in Irish nursing homes also reported a deep sense of isolation and loneliness due to restrictions placed on visiting, feeling the absence of human contact even though many used phones and computers to keep in touch ( Health Information and Quality Authority, 2020). For many, the true psychological impacts of the pandemic may be profound but may only become clear in the longer term, highlighting the need for additional research ( Hotopf *et al*., 2020).

***Mental health and well-being outcomes during the COVID-19 pandemic for people with intellectual disability and carers***. Mental health and behavioural difficulties have become more severe during the COVID-19 restrictions for some people with intellectual disability when daily routines are heavily disrupted ( Schuengel *et al*., 2020). There are also concerns that people with intellectual disability may become more vulnerable to exploitation or abuse when social support networks are no longer available ( Courtenay & Perera, 2020).

An Irish survey of individuals with intellectual disability and family members during the pandemic found that 38% of all respondents reported increased behaviours of concern, 33% increased anxiety and over half (56%) significant loneliness ( Inclusion Ireland, 2020). A small study by people with intellectual disability themselves reported that respondents with intellectual disability in Ireland found the COVID-19 lockdown period very disruptive to their usual routine including work, day services and social activities, with many reporting the frustration and emotional impact of lockdown. However, participants also spoke of their resilience and coping mechanisms ( Murphy *et al*., 2020).

These findings echo similar results from a US study, where lockdown measures were found to greatly affect access to health and educational services for people with intellectual disability with COVID-19 ( Jeste *et al*., 2020). In December 2020, a UK survey of 179 people with intellectual disability reported that, since March 2020, four out of ten respondents who previously saw their GP regularly had not seen them at all, and half of those who previously attended a day centre had not been at all in that time ( CEDAR, 2020).

The pandemic has also been challenging for caregivers, with increased risk of social isolation from service closures and disrupted social supports ( Migliaccio & Bouzigues, 2020). Informal carers of children and adults with intellectual disability reported significantly greater levels of maladaptive coping, and concerns about feeling of defeat/entrapment, anxiety and depression compared with carers of children without intellectual disability; differences increased during the pandemic and, despite having more mental health needs, carers of people with intellectual disability reported less social support ( Willner *et al.*, 2020). In an Irish survey, most carers were concerned about the declining health and well-being of the person they cared for (63%), about their own mental health and well-being (60%), and about increased challenging behaviours by their loved one (56%) ( Family Carers Ireland, 2020). Similarly, an Indian study found significant increases in caregiver strain compared to pre-pandemic levels, with a high prevalence of reported depression (62.5%), and many also reporting stress (36.4%) and anxiety (20.5%) ( Dhiman *et al*., 2020).

### Study aim

The aim of this study was to assess the impact of COVID-19 for older adults with intellectual disability in Ireland during the first wave of the pandemic and to examine rates of testing, symptoms, infection and outcomes of the disease, as well as measures taken by individuals and service providers in responding to the pandemic. It also looked at mental health impacts associated with ‘lockdown’ measures during the same period.

## Methods

### Study design and ethical approval

This was a cross-sectional survey-based study. Data was drawn from a supplementary COVID-19 questionnaire added to the fourth wave of the IDS-TILDA ( McCarron *et al*., 2020). The questionnaire was administered between May and September 2020. Prior ethical approval for IDS-TILDA, granted by the Faculty of Health Sciences Research Ethics Committee (REC) at Trinity College Dublin (TCD) and by disability service providers supporting IDS-TILDA participants, was amended to include the COVID-19 survey and approval was also granted by the National Research Ethics Committee for COVID-19-related research (NREC COVID-19).

### Population

IDS-TILDA is a longitudinal study of ageing among adults with an intellectual disability in Ireland aged 40 years and above, which completed its fourth wave of data collection in 2020, with previous waves completed in 2011, 2014 and 2017. IDS-TILDA aims to identify the principal influences on ageing in people with an intellectual disability aged 40 years and above in the Republic of Ireland. It aims to characterise and understand changes in ageing by examining healthy and successful ageing, determinants of health and longevity, and similarities or differences in ageing for those with and without intellectual disability using comparative data from TILDA for the general population. From a total sample of 739 individuals who took part in wave 4 of IDS-TILDA, 710 consented to participate in the COVID-19 survey. Table 1 provides an overview of the demographic profile of the sample.

**Table 1.** Demographic profile of the COVID-19 survey sample.

	% (n)
**Gender**	
Male	46.8 (332)
Female	53.2 (378)
**Age**	
40–49 years	18.7 (133)
50–64 years	55.1 (391)
65+ years	26.2 (186)
**Level of Intellectual Disability**	
Mild	29.7 (196)
Moderate	41.8 (276)
Severe-Profound	28.5 (188)
**Aetiology of Intellectual Disability**	
Down syndrome	19.6 (139)
Other/Unknown	80.4 (571)
**Residence Type**	
Independent/Family	17.4 (122)
Community Group Home	49.6 (348)
Residential Care	33.0 (231)
**Total**	100.0 (710)

Written consent to take part in the larger IDS-TILDA study was obtained for all participants and this was re-affirmed prior to all individual study components including the COVID-19 survey (see McCarron *et al*. (2020) for a detailed overview).

### Procedures

Nine new questions on symptoms, testing and treatment of COVID-19, as well as participants’ experiences during the crisis, were developed for the COVID-19 survey and included some open text response options ( Table 2). The survey was administered by phone or video call in one of three ways: (a) directly with the participant (13.9%, n=99); (b) with the participant and a support person (20.1%, n=143); or (c) by a proxy respondent speaking on behalf of the participant (65.9%, n=468).

**Table 2.** COVID-19 survey questions.

1) Do you/did you have any symptoms of COVID-19? a. If you do/did have symptoms, which ones do/did you have? 2) Have you been tested for COVID-19? If yes, how many times? a. If you were tested, [what] was your result? 3) Did you need to move from your usual home due to the COVID-19 crisis? If yes, what was the reason? 4) If you tested positive, and/or had symptoms, did you/your carer have a plan in place to manage the self-isolation as per COVID-19 guidelines? 5) If you tested positive, and/or had symptoms, were you able to comply with the prevention guidelines on contracting COVID-19? 6) If you tested positive and/or had symptoms, were you hospitalised? a. If admitted to hospital, how many days did you spend in hospital? 7) If admitted to hospital because of COVID-19, did your treatment require admission to intensive care? 8) Did you feel stressed/anxious about COVID-19? a. If you did feel stressed/anxious due to COVID-19, what was the reason? 9) Were there any good things about the COVID-19 period? What were they?

### Variables and data analysis

Health data including prevalence of chronic conditions (physical and mental health), and socio-demographic variables including level of intellectual disability, presence of Down syndrome and living circumstances from the larger IDS-TILDA dataset were also included in the analyses. Both univariate and bivariate descriptive analyses were performed using RStudio version 1.2.5033. Missing data were excluded from analyses. In the univariate case, numerical variables were descriptively analysed by considering their five-point summary (minimum, first quartile, median, third quartile and maximum), mean and standard deviation. Categorical variables were univariately summarised through frequencies and proportions. Bivariate descriptive analysis consisted of the cross-tabulation of two categorical variables, reporting both the frequency and proportion of each intersection. Variables used in cross-tabulations included demographic variables from Table 1, measures from the COVID-19 survey in Table 2, and health conditions outlined in Table 3. With regards to the bivariate proportions, in most cases the proportions were calculated by conditioning on the row characteristic, for example demographic characteristics. Where the bivariate relationship between a numerical and a categorical variable was described, the numerical descriptive measures mentioned in the univariate case were calculated for each group of the categorical variable.

**Table 3.** Prevalence of high-risk chronic conditions by age. BMI=body mass index; TIA=transient ischaemic attack.

Chronic Condition	<50 years %	50–69 years %	70+ years %	Total %
BMI Overweight/Obesity (n=548)	65.7	67.4	64.4	66.6
High Cholesterol (n=709)	10.5	42.8	55	38.6
Cardiovascular Disease (n=710)	12.0	34.0	58.7	33.7
Epilepsy (n=708)	22.7	31.3	30.3	29.5
Hypertension (n=709)	6.8	20.8	43.1	21.6
Arthritis (n=708)	4.5	15.2	26.6	15
Smoking (n=704)	5.3	10.6	16.7	10.5
Diabetes (n=709)	6	8.8	17.4	9.6
Lung Disease or Asthma (n=710)	5.3	8.5	12.8	8.6
Stroke or TIA (n=709)	0.8	3.2	16.5	4.8
Dementia (n=708)	0.8	4.5	6.4	4.1
Chronic Kidney Disease (n=708)	2.3	1.9	0.9	1.8
Heart Attack (n=709)	0.8	0.6	2.8	1

## Results

Table 3 illustrates the prevalence of high-risk chronic conditions reported by participant age group. This shows a clear pattern of higher prevalence with increasing age for most chronic conditions including cardiovascular disease, high cholesterol, hypertension, arthritis, smoking history, diabetes, lung disease or asthma, stroke/TIA, dementia and heart attack. Increasing morbidity with age also implies a greater risk for COVID-19 outcomes as age increases.

### COVID-19 symptoms, testing and outcomes

By the completion of data collection at the end of September 2020, nearly two-thirds (62.4%, 443/710) of participants had been tested at least once for COVID-19, with one in five of these tested more than once. The vast majority of participants who were invited for a test complied, with just 14 not consenting. Table 4 outlines findings for participants who experienced COVID-19 like symptoms, who were tested, and who tested positive. People living in residential settings had the highest rates of being tested at 84.8% (196/231), compared with 63.8% (222/348) in community group homes, and just 17.2% (21/122) of participants living in independently or with family. Participants aged 65 years and above (74.2%, 138/186) were more likely to be tested than younger participants; as were those with severe-profound intellectual disability (76.6%, 144/188) compared with mild-moderate intellectual disability; while participants with Down syndrome were less likely to the tested (49.6%, 69/139) than other participants (65.5%, 374/571).

In total, 10% (71/710) of all participants reported experiencing COVID-19-like symptoms, with higher rates reported among respondents in residential care (15.2%, 35/231), among those aged 65 years and above (12.9%, 24/186), those with severe-profound intellectual disability (12.2%, 23/188) and female respondents (11.4%, 43/378). The most common COVID-19-like symptoms reported by participants were fever (57.7%, 41/71), cough (43.7%, 31/71), fatigue (12.7%, 9/71) and shortness of breath (9.9%, 7/71).

Just 2.5% (11/443) of those tested were diagnosed with COVID-19. Of the 11 participants who tested positive, nine lived in residential care, eight were male, six had severe-profound intellectual disability and none had Down syndrome. Seven experienced symptoms and four were asymptomatic. Nine of the 11 had a history of high-risk health conditions; and three of the 11 were hospitalised, all of whom reported symptoms and had high-risk comorbidities.

**Table 4.** Participants tested for COVID-19, positive tests, and symptomatic participants.

	Symptomatic % (n) of all participants	Tested for COVID-19 % (n)	Tested Positive % (n) of those tested
**Gender**			
Male	8.4 (28)	63.6 (211)	3.8 (8)
Female	11.4 (43)	61.4 (232)	1.3 (3)
**Age**			
40–49 years	9.0 (12)	53.4 (71)	2.8 (2)
50–64 years	9.0 (35)	59.8 (234)	3.0 (7)
65+ years	12.9 (24)	74.2 (138)	1.4 (2)
**Level of Intellectual Disability**			
Mild	10.2 (20)	55.6 (109)	0.9 (1)
Moderate	8.7 (24)	60.5 (167)	1.8 (3)
Severe-Profound	12.2 (23)	76.6 (144)	4.2 (6)
**Aetiology of Intellectual Disability**			
Down syndrome	9.4 (13)	49.6 (69)	0.0 (0)
Other/Unknown	10.2 (58)	65.5 (374)	3.0 (11)
**Residence Type**			
Independent/Family	4.9 (6)	17.2 (21)	0 (0)
Community Group Home	8.6 (30)	63.8 (222)	0.9 (2)
Residential Care	15.2 (35)	84.8 (196)	4.6 (9)
**Total**	**10.0 (71)**	**62.4 (443)**	**2.5 (11)**

### Management of COVID-19 within services and other settings

A small proportion of participants (7.8%, 55/705) moved from their usual home because of the COVID-19 crisis, most commonly to isolate while waiting for test results or as a precaution (n=24), to move to a family home (n=11), or to isolate after discharge from hospital (n=7). Among participants who reported COVID-19-like symptoms or who tested positive, a large majority (78.7%, 59/75) said they had a plan in place to manage self-isolation in accordance with public health guidelines; most (61.3%, 46/75) were able to comply with prevention guidelines, but a third were unable to comply (33.3%, 25/75).

### Stress and anxiety due to COVID-19

Over half of participants (55.3%, 383/692) reported feeling stress or anxiety due to the COVID-19 crisis. Female participants (57.8%, 214/370), those aged 40–49 years (59.5%, 78/131), those with mild intellectual disability (63.9%, 122/191), and those living in independent or family residences (59.5%, 72/121) were the most likely to report stress/anxiety due to COVID-19. Rates of stress/anxiety reported for participants with severe-profound intellectual disability (36.8%, 68/185) were considerably lower than those with mild-moderate intellectual disability, as was the case for people in residential care (49.8%, 111/223) compared with other types of accommodation.

The relationship between pre-existing anxiety and reported COVID-19 related stress/anxiety was explored. We found that participants who reported COVID-19 related stress/anxiety had higher rates of pre-existing anxiety (26.7%, 54/202) than those who did not report COVID-19 stress/anxiety (12.6%, 14/111).

Causes of stress/anxiety related to COVID-19 were also explored. The most commonly reported cause of COVID-19 stress/anxiety was not being able to do one’s usual activities, reported by 79.1% (303/383) of participants. Other common causes of stress/anxiety included not seeing family (47.0%, 180/383), not seeing friends (45.4%, 174/383), loneliness (26.9%, 103/383), isolation (15.9%, 61/383), and fear of getting COVID-19 (15.7%, 60/383). These most common causes of stress/anxiety were further analysed by residential setting. A smaller majority of participants living in residential care (69.4%, 77/111) reported stress/anxiety due to being unable to do their usual activities compared with participants in independent/family residences (84.7%, 61/72) and community group homes (82.2%, 162/197). While fewer participants in independent/family settings (27.8%, 20/72) reported stress/anxiety caused by not seeing family, compared with those living in community group homes (55.3%, 109/197) and residential settings (44.1%, 49/111); considerably more participants in independent/family settings (61.1%, 44/72) were stressed/anxious due to not seeing their friends, compared to those in community group homes (45.2%, 89/197) and residential settings (34.2%, 38/111). Similarly, more participants in independent/family homes (38.9%, 28/72) reported loneliness as a cause of stress/anxiety during the COVID-19 crisis, compared with residents of community group homes (25.9%, 51/197) and residential care (20.7%, 23/111).

Participants with severe-profound intellectual disability and those living in residential care reported substantially lower rates of stress/anxiety. Further analysis showed that participants who self-reported without support reported much higher rates of stress/anxiety due to COVID-19 (69.4%, 68/98) compared with reports from proxy respondents (49.7%, 227/457). A third group who self-reported with some support reported rates of COVID-19 stress/anxiety closer to the self-reporters (64.2%, 88/137).

### Positive aspects of the COVID-19 period

The most commonly reported positive aspects of the COVID-19 period were: the opportunity to engage in new/more activities (41.2%, 157/381); the opportunity for more rest and relaxation (36.0%, 137/381); more time and/or better relations with staff (26.0%, 99/381); and the opportunity to use technology to communicate with others (13.6%, 52/381). Almost 60% of participants (58.3%, 381/654) reported that there were some good things about the COVID-19 period, with more female participants identifying positive aspects (60.3%, 207/343) than males (55.9%, 174/311). Rates of positivity increased marginally with age, from 55.5% (61/110) for participants aged 40–49 years to 58.0% (210/362) for those aged 50–64 years and was highest for the oldest group aged 65+ years (60.4%, 110/182). Participants with moderate intellectual disability (55.8%, 140/251) had slightly lower rates than others with mild (61.1%, 110/180) and severe-profound intellectual disability (60.8%, 107/176). More residents of community group homes (61.3%, 198/323) identified good things about the COVID-19 period compared with those living in independent/family settings (50.9%, 55/108), and with participants from residential care settings (57.5%, 123/214).

Many participants who responded to the questions about stress/anxiety and good things about the COVID-19 period (31.4%, 203/646) reported that they had experienced both stress/anxiety and positive aspects. For example, 84 individuals identified not being able to do their usual activities as a source of stress/anxiety, yet also identified the opportunity to engage in new/more activities as a positive aspect of the COVID-19 period. Furthermore, several responses to the question on good things about the COVID-19 period were ambivalent, often highlighting both positive and negative aspects. These ambivalent responses tended to come from proxy respondents rather than self-reporting participants, for example:

 

*“…increasing activities such as arts and crafts, colouring. This was not done in the house before COVID and he has enjoyed this activity, although missing work”*

*“…he was happy enough, quite contented. Recently he started looking forward to returning to his job in a hotel and his day services which are starting up again soon.”*

*“More equipment such as sports equipment and gardening items were made available, but he did not engage with these.”*

*“…would have liked the lie ins but misses his day service.”*

*“…gets more rest not having to be up early to get transport to day services, albeit lack of structure/routine did affect her mental health”*

### Impact of COVID-19 on respondents with Down syndrome

The literature review identified that Down syndrome may be a particular risk for adverse outcomes of COVID-19. Of the 710 participants with intellectual disability in the current study, 139 individuals had Down syndrome.

The average age of participants with Down syndrome was 52.0 years, compared with 59.9 years for other participants; and while almost a third of participants without Down syndrome (31.5%, 180/571) were aged 65 years or older, just 4.3% (6/139) of participants with Down syndrome were in this oldest age group. Notable differences were also observed with respect to residential setting. The most common residential setting for both groups was community group homes (52.9% of participants with Down syndrome; 48.8% of other participants). But more participants with Down syndrome lived in independent/family settings (24.3% compared with 15.8%) and fewer lived in residential care settings (22.8% compared with 35.4%)

Similar rates of obesity/overweight were observed in participants with Down syndrome compared with other participants (68.2% and 66.2% respectively). However, participants with Down syndrome had considerably lower prevalence of several high-risk conditions associated with COVID-19, including cardiovascular disease (26.6% compared with 37.7%), high cholesterol (24.5% compared with 42.1%), epilepsy (13.7% compared with 33.4%), hypertension (5.0% compared with 25.6%), diabetes (3.6% compared with 11.1%) and a history of smoking (2.2% compared with 12.4%). Participants with Down syndrome did have substantially higher prevalence of dementia (12.2%) compared with participants with intellectual disability from other aetiologies (2.1%). With regard to mental health, there was little difference between participants with and without Down syndrome in rates of depression using the Glasgow Depression Scale (6.9% compared with 7.2% on the self-reported scale; and 9.9% compared with 7.2% on the carer-reported scale); however, participants with Down syndrome had lower rates of anxiety on the Glasgow Anxiety Scale compared with other participants (15.8% and 22.9% respectively).

***Comparing outcomes of COVID-19***. With regard to COVID-19-like symptoms, a slightly smaller proportion of participants with Down syndrome reported such symptoms (9.4%, 13/139) compared with other participants (11.6%, 57/493). Of the 13 participants with Down syndrome who reported symptoms, two were hospitalised but neither tested positive for COVID-19. Fewer participants with Down syndrome were tested for COVID-19 (50.0%, 69/138) compared with participants without Down syndrome (69.6%, 339/487). None of those participants with Down syndrome who were tested for COVID-19 returned a positive test.

## Discussion

This paper examined the experience in Ireland of the first wave of COVID-19 among older adults with intellectual disability. Despite similar risk factors, as compared to the general older population, data presented here indicated people ageing with intellectual disability were, at least during this initial virus wave, far less severely impacted by COVID-19 in terms of rates of infection, hospitalisation and mortality – with no deaths among our nationally representative sample. The data also suggests that people with intellectual disability have faced significant challenges caused by disruption to their normal routines and social connections, but have responded with commendable adaptability and resilience. However, this data provides a time-limited snapshot of the experiences of this population during the first wave of the COVID-19 pandemic in Ireland, up to the end of September 2020. As such, this paper provides a baseline against which the ongoing experiences and long-term impacts of COVID-19 for older adults with intellectual disability may be measured.

### Exposure and infection risk for older adults with intellectual disability

Almost two-thirds of IDS-TILDA participants had been tested at the time of data collection, rising to six out of seven participants in residential care, and an overall infection rate of 2.5%, compared with a national positivity rate of 3.4% at the end of September 2020. Rates of testing here were much higher than in the general population (based on 1.2 million tests completed nationally, equating to less than a quarter of the total population considering that some were tested multiple times). A positive test rate of 4.6% among IDS-TILDA participants in residential care was lower than high rates of contagion reported in nursing homes; 22% of all cases and 56% of all deaths were reported in these settings ( COVID-19 Nursing Homes Expert Panel, 2020). Further longitudinal data is needed to ascertain if these low rates were sustained and if so, to establish what contributed to these comparatively low infection and mortality rates within the intellectual disability population to date, despite their high-risk characteristics for both infection and adverse outcomes of COVID-19.

### COVID-19 Infection and outcomes among IDS-TILDA participants

With reported COVID-19 mortality rates in the general population of almost 30% over the age of 80 years, and rising rapidly after 50 years from less than 1.1% under 50, our findings of no deaths are to be welcomed. However, after similar initial reports of lower mortality rates among people with intellectual disability in other countries, more recent international studies have reported mortality of 3–8 times higher for people with intellectual disability ( Public Health England, 2020a; Watkins, 2020), and 10 times higher for people with Down syndrome ( Clift *et al*., 2020). Age of severe impact also appears lower compared with the general population, with average age of death reported as 64 years for people with intellectual disability ( Perera *et al*., 2020) and 51 years for people with Down syndrome ( Hüls *et al*., 2020).

We recorded no infections or deaths among participants with Down syndrome, despite longitudinal health data showing high rates of known risk factors including obesity, epilepsy and dementia. While the number of infections reported here were low, there were observations of more infection among participants with severe and profound intellectual disability and among those in residential care settings (who tend to be older with more chronic conditions and to be non-ambulatory). These observations suggest that, moving forward, attention should be paid to age, health status and level of intellectual disability. 

In comparison with the general older population in Ireland reported by TILDA ( Hernández *et al*., 2020), prevalence rates for known risk factors were lower in people with intellectual disability for cardiovascular disease (TILDA prevalence was 44.7% at 50–69 years compared with 34.0% here; and 66.6% at 70+ years compared with 58.7% here), hypertension (TILDA prevalence was 42.9% at 50–69 years compared with 20.8% here; and 61.1% at 70+ years compared with 43.1% here) and chronic kidney disease (TILDA prevalence was 5.7% at 50–69 years compared with 1.9% here; and 28% at 70+ years compared with 0.9% here). Diabetes was more prevalent in the general population for people aged 50–69 years (10.5% compared with 8.8% here) but less prevalent in those aged 70+ years (14.9% compared with 17.4% here). There was a higher overall prevalence in the general population for high cholesterol (58.5% compared with 38.6% here), arthritis (45.6% compared with 15.0% here), lung disease/asthma (18.3% compared with 8.6% here) and heart attack (6.2% compared with 1.0% here).

### Impact on social inclusion, mental health and well-being

Our data confirmed that, during the first wave of the COVID-19 pandemic in Ireland, more than half of participants experienced some COVID-19 related stress or anxiety, and that higher rates of COVID-19 related stress/anxiety were associated with higher rates of pre-existing anxiety, as identified in literature ( Asmundson *et al*., 2020). Over half of all participants reported COVID-19 stress/anxiety, which is considerably higher than rates of between 16–28% reported in the general population ( Moghanibashi-Mansourieh, 2020; Rajkumar, 2020) though below findings of 65% perceived stress for the general older Irish population reported by TILDA from data collected later in the pandemic ( De Looze & McDowell, 2021). However, a similar proportion of our participants also reported positive aspects about this COVID-19 period, with many reporting both positive and negative experiences. The impact of prolonged COVID-19 restrictions through subsequent waves of infection must be monitored longitudinally with particular focus on additional stress for those with pre-existing mental health conditions.

There were notable differences in rates and types of stress/anxiety reported by particular groups, with higher rates among self-reporting participants compared with proxy respondents, which raises different possible explanations. For example, the most independent participants who were able to speak for themselves may have felt the greatest sense of restriction from COVID-19; or alternately, the extent of stress/anxiety for participants who relied on proxy respondents may have been underreported. Restrictions to social connections varied among participants, as did their feelings about connections lost, while increased contact with staff during the restrictions may be more valued by others. Also, the use of technology as a potential mitigating factor in social contacts is noteworthy. IDS-TILDA has previously highlighted the persistent digital divide experienced by people with intellectual disability ( McCausland *et al*., 2017). In Wave 4, the data suggests a noticeable increase in access to and use of technology by this population ( McCarron *et al*., 2020; McCausland *et al*., 2021). It is critical that any such gains made during this period are not lost and that support for digital inclusion is maintained.

With more participants living in independent/family residences reporting that they missed friends and felt lonely compared with other residential settings, particular attention may be needed for this cohort. IDS-TILDA previously highlighted that more people living in community group homes and residential care identified co-resident peers and support staff as their friends ( McCausland *et al*., 2020). This implies that many may continue seeing these friends even during full COVID-19 lockdown, while more independent participants are relatively cut off from their non-resident, non-staff friends. Furthermore, restricted access to technology and lack of personal transportation may have exacerbated the isolation felt by the most independent during COVID-19. Additionally, fewer participants in independent/family residences missed family compared with residents in other settings. Again, these risks must be monitored longitudinally as the cumulative effect of ongoing social restrictions may necessitate a reorganisation of supports to those most at risk.

IDS-TILDA participants were aged 40 years and older and many from this age reported multiple chronic health conditions. The low infection and mortality rates reported here perhaps justifies the measures taken in Ireland to safeguard people with ID and others during the first wave of infection. However, additional longitudinal data is needed to give a clearer understanding of longer-term consequences for well-being of safeguarding measures and how these balance against infection risks and outcomes.

### Comparing models of care with the general older population

Lower rates of COVID-19 infection reported here compared with the general older population, particularly in residential care facilities, raise important questions about the underlying reasons for such differences. Reviews conducted by the Health Information and Quality Authority (HIQA) ( Health Information and Quality Authority, 2020) and the Nursing Home Expert Group of nursing homes in Ireland during the COVID-19 crisis ( COVID-19 Nursing Homes Expert Panel, 2020) offered insight as to how nursing homes may have failed or succeeded in preventing infection outbreaks. This included questions around clinical oversight and governance, staff skills and skills-mix; and highlighting high rates of testing, good planning and procedures, and adherence to public health guidelines as key factors in preventing outbreaks within facilities. Anecdotal reports from services (including, for example, during webinars organised by the Trinity Centre for Ageing and Intellectual Disability (TCAID) during the pandemic ^1^), confirmed in IDS-TILDA data, suggest there were similar strategies by services caring for people with intellectual disability. This included high levels of planning for isolation and successful implementation of isolation plans. This may help explain the lower levels of infection and mortality found here.

The HIQA and the Nursing Home Expert Group reviews questioned the dominant model of care for older people in Ireland, which tends towards placing older people with additional supports needs in large nursing homes, ahead of options to support them in their own homes ( COVID-19 Nursing Homes Expert Panel, 2020; Health Information and Quality Authority, 2020). This model of moving older people to large centres contrasts with the policy focus on de-congregation of people with intellectual disability living in large institutions to smaller community-based residences, which has resulted in the closure of larger residential units ( Health Service Executive, 2011). Suggestions that transmission of COVID-19 is associated with size of care facility remains tentative; given numbers reported here are too small to draw conclusions and that most studies internationally found that size of care facility was not significant once other factors were controlled for including extent of the outbreak within the surrounding community or assessed quality of facilities ( Bui *et al*., 2020; He *et al*., 2020; Shi *et al*., 2020). Therefore, additional research on the role of size and type of setting is required to explore this in an Irish context.

## Conclusions

Our findings suggest that a concerted effort by people with intellectual disability, their families and service providers – including widespread testing, good planning and adherence to public health guidelines – may have helped to avert the worst impacts of COVID-19 during the first wave of infection in Ireland. It may be that the general older sector can learn from approaches adopted by the intellectual disability sector. More recent stark findings internationally, including much higher rates of COVID-19 mortality among people with intellectual disability, suggest that difficulties may arise in a prolonged fight through multiple waves of COVID-19. While COVID-19 remains a continuing and even growing threat in society, this older population with histories of high-risk health conditions remains vulnerable and will require continued safeguarding from the disease.

Both our findings and the international literature suggest that screening for high-risk comorbidities or frailty more so than chronological age should form the basis of assessment for COVID-19 risk in people with intellectual disability. This is especially important as the rollout of vaccines against the disease is commencing and groups are prioritised. The threat to mental health and well-being presented by the continued curtailment of normal routines, service closures and social inclusion also requires ongoing efforts by services to provide flexible and responsive support to people with intellectual disability and their families. Finally, as subsequent waves of COVID-19 infections occur, the longitudinal assessment of how the pandemic has had an impact on persons ageing with intellectual disability will be critical to responding to emerging needs and any required redesigning of the support infrastructure in the future. Our planned repeated data collection will provide critical data to inform a model of recovery from the COVID-19 pandemic for people with an intellectual disability.

## Data availability

### Underlying data

Approval for data sharing was not sought at ethics approval stage nor was it included in the study information and consent forms provided to participants. The anonymised underlying data for this paper is available in a restricted format. Access to data which could potentially pose a risk to the confidentiality of IDS-TILDA participants has been withheld following assessment of sample size, cell counts and the data context.

Anonymised data and study documentation may be accessed through the Irish Social Science Data Archive (ISSDA) at https://www.ucd.ie/issda/data/ids-tilda/. To access the data, please complete a ISSDA Data Request Form for Research Purposes, sign it, and send it to ISSDA by email.

For teaching purposes, please complete the ISSDA Data Request Form for Teaching Purposes, and follow the procedures, as above. Teaching requests are approved on a once-off module/workshop basis. Subsequent occurrences of the module/workshop require a new teaching request form.

Data will be disseminated on receipt of a fully completed, signed form. Incomplete or unsigned forms will be returned to the data requester for completion.

## Footnotes

^1^ https://www.tcd.ie/tcaid/about/webinars.php
