# Supplementary material for: The impact of coronavirus disease 2019 (COVID-19) on older adults with an intellectual disability during the first wave of the pandemic in Ireland
Source: HRB Open Res. 2021 Dec 13;4:93. Originally published 2021 Aug 19. [Version 2] doi: 10.12688/hrbopenres.13238.2 (PMC8772523; doi:10.12688/hrbopenres.13238.2)
Supplement: Peer review history

# Peer review: Reviewer response for version 2

**DOI:** 10.21956/hrbopenres.14687.r31080

**Published:** 2022-01-19

**Authors:** Meaghan Edwards

**Affiliations:** 1 Rehabilitation and Disability Studies, Cumming School of Medicine, University of Calgary, Calgary, AB, Canada

The article is much improved with the inclusion of a discussion of the limitations of proxy reports. It is valuable to point out that the inequities and lack of inclusion and access already experienced by this population were exacerbated by the circumstances of the pandemic.

Is the work clearly and accurately presented and does it cite the current literature?

Yes

If applicable, is the statistical analysis and its interpretation appropriate?

Yes

Are all the source data underlying the results available to ensure full reproducibility?

Partly

Is the study design appropriate and is the work technically sound?

Partly

Are the conclusions drawn adequately supported by the results?

Partly

Are sufficient details of methods and analysis provided to allow replication by others?

Partly

Reviewer Expertise:

Disability studies, community-based research, marginalization and vulnerable populations

I confirm that I have read this submission and believe that I have an appropriate level of expertise to confirm that it is of an acceptable scientific standard.

# Peer review: Reviewer response for version 2

**DOI:** 10.21956/hrbopenres.14687.r31079

**Published:** 2022-01-05

**Authors:** Jacqueline M. McGinley, Shamrock Wumpini Osman

**Affiliations:** 1 College of Community & Public Affairs, Binghamton University, Binghamton, NY, USA; 2 Department of Social Work, College of Community & Public Affairs, Binghamton University, Binghamton, NY, USA

No further comments at this time. We appreciate the authors' revisions and responses to our original comments.

Is the work clearly and accurately presented and does it cite the current literature?

Yes

If applicable, is the statistical analysis and its interpretation appropriate?

Yes

Are all the source data underlying the results available to ensure full reproducibility?

Yes

Is the study design appropriate and is the work technically sound?

Yes

Are the conclusions drawn adequately supported by the results?

Yes

Are sufficient details of methods and analysis provided to allow replication by others?

Partly

Reviewer Expertise:

Intellectual and Developmental Disabilities; Aging Populations and their Families; End-of-life Care and Bereavement

We confirm that we have read this submission and believe that we have an appropriate level of expertise to confirm that it is of an acceptable scientific standard.

# Peer review: Reviewer response for version 1

**DOI:** 10.21956/hrbopenres.14401.r30088

**Published:** 2021-09-27

**Authors:** Meaghan Edwards

**Affiliations:** 1 Rehabilitation and Disability Studies, Cumming School of Medicine, University of Calgary, Calgary, AB, Canada

Risk factors and COVID-19-would be helpful to indicate one or two reasons for the increased risk in congregated care settings close to the beginning. Also important to suggest why people with ID are at higher risk (low paying, unsafe jobs, congregated living, public transportation, physical/genetic factors, etc.)

 This is an interesting and well-organized paper and I enjoyed reading it. 

 All of my partly answers refer to the following:

- I think the issue of proxy needs to be examined in more detail-a significant portion of the sample answered in this way and especially for the mental health questions, I think this needs more description. When answering by proxy one cannot be sure of the reasons for anxiety-it could very well be related to other things including relational difficulties with the very support person being interviewed. This is addressed in the discussion but not clearly enough-it is a serious limitation and the language of "participants reported" is slightly jarring when one considers that these participants were not the people with disabilities many times.
- There is a significant risk when this proxy is a staff member. Many families here in Canada were very concerned that instances of abuse and "silencing" of their family members would increase during COVID when people were unable to connect directly with the family. I would like to see the authors write something about this in limitations and in the body of the study itself. 
- Otherwise, I think it is a worthy study!

Is the work clearly and accurately presented and does it cite the current literature?

Yes

If applicable, is the statistical analysis and its interpretation appropriate?

Yes

Are all the source data underlying the results available to ensure full reproducibility?

Partly

Is the study design appropriate and is the work technically sound?

Partly

Are the conclusions drawn adequately supported by the results?

Partly

Are sufficient details of methods and analysis provided to allow replication by others?

Partly

Reviewer Expertise:

Disability studies, community-based research, marginalization and vulnerable populations

I confirm that I have read this submission and believe that I have an appropriate level of expertise to confirm that it is of an acceptable scientific standard, however I have significant reservations, as outlined above.

# Peer review: Reviewer response for version 1

**DOI:** 10.21956/hrbopenres.14401.r30086

**Published:** 2021-09-06

**Authors:** Jacqueline M. McGinley, Shamrock Wumpini Osman

**Affiliations:** 1 Department of Social Work, College of Community & Public Affairs, Binghamton University, Binghamton, NY, USA; 2 College of Community & Public Affairs, Binghamton University, Binghamton, NY, USA

Overall, we found this article to have a number of merits, particularly in terms of its important contributions to the emergent body of literature on the experiences of people with intellectual disabilities throughout the COVID-19 pandemic. We particularly appreciated that the authors attended to the implications of the pandemic on individuals’ health, mental health, and overall well being. We also found the section on the “Positive Aspects of the COVID-19 period” to be an important contribution, as it highlighted areas for consideration and themes for replication in a post-pandemic world. 

 We rated this manuscript as “Approved with Reservations” because we have several minor recommendations and one substantive recommendation, which we would like to invite the authors to consider:

1. Of greatest significance, we would ask that the authors consider including additional details in their “Methods” section (perhaps in the “Variables and data analysis” subsection) regarding their approach to qualitative data analysis. At present, only their quantitative methods are described. The instrument provided appears to include open-ended questions, and the “Results” section seems to report on qualitative response. It would be helpful to know, for example, how/if qualitative responses were coded, collapsed, and synthesized.
2. We may have overlooked this within the article, but we could not determine the exact time period that was referred to as the “first wave”. It may be helpful to international readers and may support comparisons more readily if the exact dates of the first wave were provided in-text.   
3. The Centers for Disease Control and Prevention (CDC, 2021) has just issued new guidance on COVID-19 and people with intellectual disabilities. This reference might replace the citation on p. 3, par. 4. http://www.advancingstates.org/hcbs/article/centers-disease-control-and-prevention-cdc
4. On p. 4, par. 5 (begins “Studies conducted early in the pandemic…”), the paragraph relates to heath care professionals and, while reporting on an important and compelling study, does not appear related to it section heading, “Outcomes of COVID-19 infections for people with intellectual disability”. We recommend either deleting the paragraph or moving it elsewhere in the manuscript.
5. The section entitled, “Mental health and well-being outcomes during the COVID-19 pandemic for people with intellectual disabilities and carers” describes the available literature on the experiences of children, young adults, and carers. Because the study results relate primarily to people who are 40 years or older, we wondered If there has been any literature to date that could be cited here that involves the experiences of those who are among the age cohorts represented in the original research.
6. On p.1, par.1 (section entitled, "Outcome of COVID-19 infection for people with intellectual disability"), the authors describe some interesting data on mortality rates. The article reports 11% mortality rate among people with intellectual disability in Netherland and higher rates among adults with intellectual disability and developmental disabilities in the US (18-75). We wondered if the authors might provide the exact mortality rates in the United States; this will enable the readers to better understand this point of comparison. 

Is the work clearly and accurately presented and does it cite the current literature?

Yes

If applicable, is the statistical analysis and its interpretation appropriate?

Yes

Are all the source data underlying the results available to ensure full reproducibility?

Yes

Is the study design appropriate and is the work technically sound?

Yes

Are the conclusions drawn adequately supported by the results?

Yes

Are sufficient details of methods and analysis provided to allow replication by others?

Partly

Reviewer Expertise:

Intellectual and Developmental Disabilities; Aging Populations and their Families; End-of-life Care and Bereavement

We confirm that we have read this submission and believe that we have an appropriate level of expertise to confirm that it is of an acceptable scientific standard, however we have significant reservations, as outlined above.
